# Wearable Multi-Frequency and Multi-Segment Bioelectrical Impedance Spectroscopy for Unobtrusively Tracking Body Fluid Shifts during Physical Activity in Real-Field Applications: A Preliminary Study

**DOI:** 10.3390/s16050673

**Published:** 2016-05-11

**Authors:** Federica Villa, Alessandro Magnani, Martina A. Maggioni, Alexander Stahn, Susanna Rampichini, Giampiero Merati, Paolo Castiglioni

**Affiliations:** 1 Dipartimento di Elettronica, Informazione e Bioingegneria, Politecnico di Milano, Milan 20133, Italy; federica.villa@polimi.it (F.V.); alessandro.magnani@polimi.it (A.M.); 2 Center for Space Medicine and Extreme Environments, Charité-University Medicine Berlin, Berlin 10117, Germany; martina.maggioni@charite.de or martina.maggioni@unimi.it (M.A.M.); Alexander.Stahn@charite.de (A.S.); 3 Department of Biomedical Sciences for Health, Università degli Studi di Milano, Milan 20133, Italy; susanna.rampichini@unimi.it (S.R.); giampiero.merati@unimi.it (G.M.); 4 IRCCS Fondazione Don C. Gnocchi, Milan 20148, Italy

**Keywords:** exercise, blood shift, body composition, electrical impedance

## Abstract

Bioelectrical Impedance Spectroscopy (BIS) allows assessing the composition of body districts noninvasively and quickly, potentially providing important physiological/clinical information. However, neither portable commercial instruments nor more advanced wearable prototypes simultaneously satisfy the demanding needs of unobtrusively tracking body fluid shifts in different segments simultaneously, over a broad frequency range, for long periods and with high measurements rate. These needs are often required to evaluate exercise tests in sports or rehabilitation medicine, or to assess gravitational stresses in aerospace medicine. Therefore, the aim of this work is to present a new wearable prototype for monitoring multi-segment and multi-frequency BIS unobtrusively over long periods. Our prototype guarantees low weight, small size and low power consumption. An analog board with current-injecting and voltage-sensing electrodes across three body segments interfaces a digital board that generates square-wave current stimuli and computes impedance at 10 frequencies from 1 to 796 kHz. To evaluate the information derivable from our device, we monitored the BIS of three body segments in a volunteer before, during and after physical exercise and postural shift. We show that it can describe the dynamics of exercise-induced changes and the effect of a sit-to-stand maneuver in active and inactive muscular districts separately and simultaneously.

## 1. Introduction

Bioelectrical Impedance Spectroscopy (BIS) is a non-invasive method that allows fast and economic evaluations of total body composition and individual body segments [1,2]. BIS injects a small oscillatory current into a body region of interest at different frequencies, *f*, and provides the magnitude and phase of the corresponding electrical impedance, Z*(f)*, by measuring the voltage drop across the segment. Several characteristics of body tissues such as volume and conductivity of intra- and extra-cellular fluids as well as cell membrane capacitance may affect the electrical impedance differently at distinctive frequencies. Therefore, BIS may potentially provide useful information for sports medicine and aerospace medicine studies or for rehabilitation protocols, including the assessment of hydration status [3], exercise-induced loss of body fluids [4], fluid shifts between body regions following postural changes [5], edema formation in the lungs [6] or limbs [7], fat-free and fat masses [8], muscle volumes [9] and changes of muscle properties after strenuous exercise [10], muscle disorders [11], as well as estimations of maximal oxygen uptake [12,13].

Most of the above-cited applications require the monitoring of the Z*(f)* spectrum over a wide range of frequencies at a high sampling rate and for different body segments simultaneously. In addition, the hardware should (1) be miniaturized; (2) require minimal power consumption; and (3) promote mobile and unobtrusive monitoring outside the laboratory. To our knowledge, neither commercially available BIS instruments (see Table S1 of the online supplement) nor more advanced wearable prototypes proposed in the recent literature [14,15] meet all these requirements. In fact, commercial off-the-shelf bioimpedance devices basically suffer from some of the following major limitations: (1) they are rather bulky and limited to laboratory/clinical settings; (2) they generally employ a single electrode set-up and model the human body as a single cylinder, preventing the possibility to distinguish fluid distribution between different body segments simultaneously; or (3) when they provide measures on more than one body segment, it is not possible to select segments different from those predefined by the system (e.g., limbs and trunk); and (4) many of them do not employ the principle of bioimpedance spectroscopy, but typically operate a single frequency (e.g., 50 kHz), limiting their use for differentiating intra- and extra-cellular water. These are considerable drawbacks for various applications in sports medicine that require the continuous monitoring of impedance changes in specific body segments without interfering movements, or the assessment of the hydration level during prolonged physical exercise, such as during a marathon run. Similarly, rehabilitation medicine may require monitoring BIS in selected body segments for therapeutic interventions. This includes, for example, free-moving patients wearing compression socks to prevent deep venous thrombosis, or patients with peripheral artery disease exercising the lower limbs, when echographic blood flow measurements are not feasible. In addition, aerospace medicine applications may necessitate continuous long-term evaluations of BIS with minimal interferences of other activities. Such recordings could assess blood pooling in the lower extremities of jet-pilots exposed to changing gravity forces, or fluid shifts concomitant with weightlessness and/or artificial gravity in astronauts. In all these cases, unobtrusive, wearable BIS systems, capable of long-term recordings, could provide important physiological and clinical information.

We have recently shown that a Digital Signal Processor (DSP) can generate the proper waveforms of the stimulation currents and elaborate the measured voltages over the frequency range requested for BIS studies [16]. Since the DSP can be integrated in a miniaturized and light-weight BIS system with very low power consumption, this paved the way for a new class of wearable BIS instruments that may allow the unobtrusive long-term monitoring of different body segments simultaneously over a broad range of frequencies. Based on the DSP approach, we have developed a novel BIS prototype that aims to provide a wearable, not just portable, multi-segmental and multi-frequency device with a built-in triaxial accelerometer. This device allows studying the relationship between movements, position and body impedance for hours with up to 50 impedance spectra per second over a broad range of frequencies. The significant impact of this technology can be traced to the following advantages: it is wearable, it enables long-term continuous (online) monitoring, it is based on a segmental approach and it implements multifrequency spectroscopy, potentially allowing intra- and extra-cellular water assessment. These characteristics are not met by any of the devices commercially available.

The aim of the present work was therefore (1) to describe a wearable prototype for monitoring BIS over different body segments without interfering with the subject movements and (2) to assess the feasibility of this prototype to quantify impedance changes associated with fluid shifts in three body segments concomitant with physical exercise as well as postural changes. 

## 2. Materials and Methods 

### 2.1. Description of the BIS Device 

**Figure 1:**
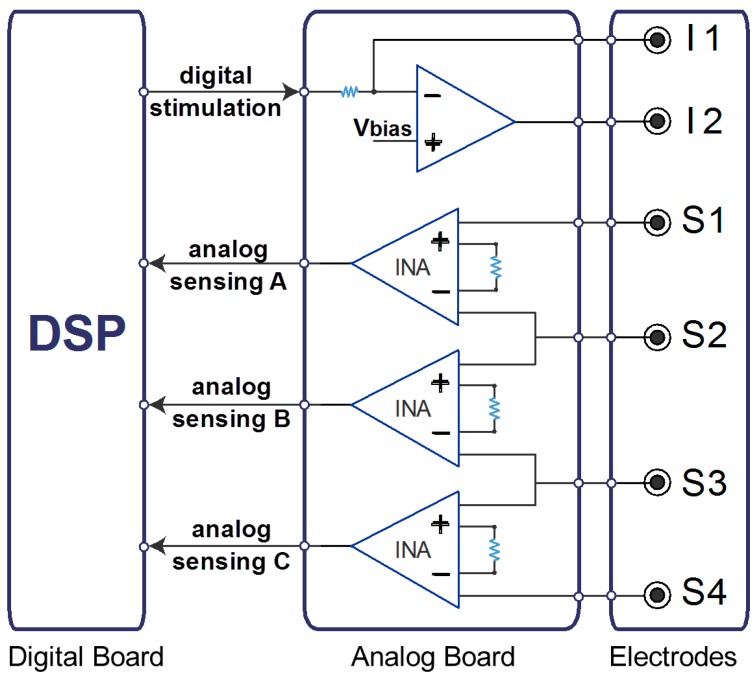
Scheme of our BIS system consisting of a digital board (**left**) with a DSP that generates the periodic stimulation waveforms and receives three sensed voltages for Fourier analysis; an analog board (**middle**) with a transconductance amplifier to produce the stimulation current and three instrumentation amplifiers for reading voltage drops across body segments; and an electrode set-up (**right**) consisting of two injecting electrodes (I1, I2) and four sensing electrodes (S1–S4).

Our BIS system comprises two boards: a digital board with a DSP (Texas Instrument C2000 “Piccolo family”, 80 MHz clock, Texas Instruments, Dallas, TX, USA) and a custom analog board (Figure 1). The DSP generates the stimulus waveforms, samples and digitalizes the voltage across three body segments with an internal 12 bit Analog to Digital Converter (ADC) and computes the magnitude and phase of *Z(f)*. The analog board interfaces the DSP with the electrodes. In particular, the analog board mounts a transconductance amplifier connected to two injecting electrodes, I1 and I2. It also contains a number of instrumentation amplifiers (INAs). Each INA reads the voltage across a specific body segment by means of a couple of electrodes. Since each couple of INAs shares one of the two electrodes, the number of required sensing electrodes is *N* + 1 if *N* is the number of INAs, corresponding to the number of body segments that can be simultaneously measured. The presented prototype was designed to evaluate up to three segments simultaneously, and therefore the analog board mounts three INAs employing four sensing electrodes. If specific applications require it, the modular design allows upgrading the analog board mounting a greater number of INAs. 

In order to minimize the power consumption, size and cost of the device, the stimulation waveform for assessing Z*(f)* at a given frequency *f*_0_ is not a sinusoid with frequency *f*_0_, as in commercially available BIS devices, but a digital square wave with fundamental frequency *f*_0_. In fact, the DSP can generate square waves more easily than sinusoids, and the use of square wave stimulations allowed us to employ an inexpensive microcontroller with low power consumption. 

**Figure 2:**
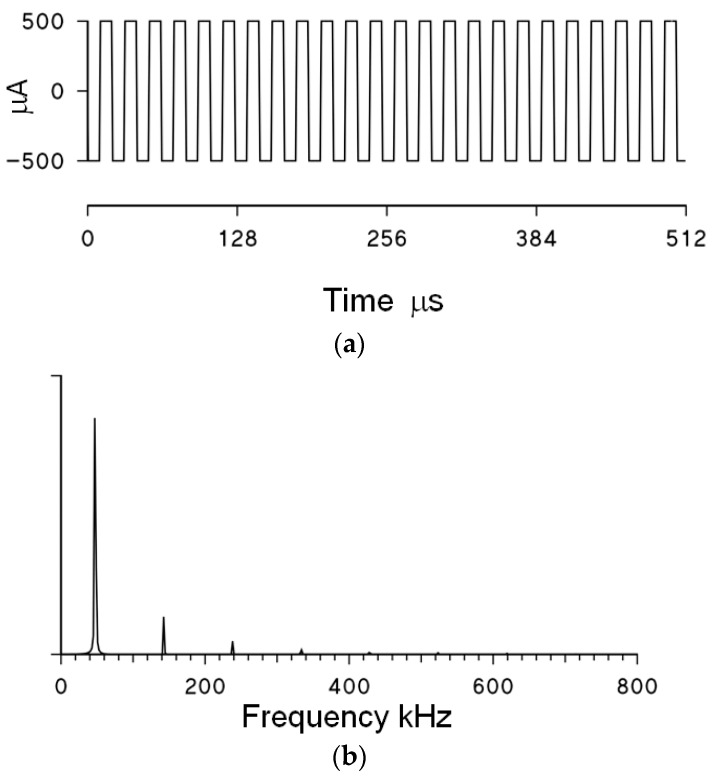
(**a**) Example of stimulation current at *f*_0_ = 48 kHz (for clarity, only a segment of the original 5-ms-long train of square waves is shown); (**b**) Modulus of the corresponding FFT spectrum; the Fourier spectrum shows a main peak at the fundamental frequency *f*_0_, and minor peaks at higher harmonics (note that the amplitude of even harmonics is zero for the square wave). Only the Fourier component at *f*_0_ is considered for the estimation of Z*(f)*.

Therefore, the DSP measures Z*(f)* at the selected frequency *f*_0_ by generating a 5 ms train of square waves with *f*_0_ as the first harmonic. The transconductance amplifier transforms the square waves in a stimulation current of amplitude ±500 μA. The ADC generates a series of 1024 samples from each analog, sensing input in real time, and the DSP calculates the corresponding fast Fourier transform (FFT). The DSP also implements a calibration procedure to compute the body impedance, independently from the system stray capacitances and resistances. Since a square wave is used, the Fourier transform is characterized not only by the main component at *f*_0_, but also by higher harmonics at odd multiples of *f*_0_ (Figure 2). The DSP extracts the modulus and phase of Z*(f)* at the fundamental frequency of the square wave only. The ADC sampling rate is 2.3 MHz and the stimulation fundamental frequencies *f*_0_ are properly selected to avoid aliasing effects at *f*_0_.

The size and number of electrodes are a critical issue for the usability of mobile, multi-segmental BIS devices. Large-band electrodes are generally used in impedance applications, because of their low impedance and because they present only minor current constriction effects, two factors that may influence Z*(f)*. Standard disk electrodes such as those typically employed for electrocardiogram recordings are preferable for their smaller size. However, we have previously shown that the impedance of small disk electrodes cannot be considered negligible at relatively low frequencies (about 10 kHz) when bipolar or tripolar electrode set-ups are employed [17]. In contrast, by using a tetrapolar electrode arrangement (*i.e.*, by separating the electrodes for injecting the stimulation current from those used for sensing the response voltage), measures of Z*(f)* are in practice independent of the electrode impedance [17]. This allows using small disk electrodes instead of larger band electrodes. The effect of constriction zones under the injecting electrodes can be minimized by placing the proximal sensing electrodes not too close to the distal injecting electrodes. The disadvantage of the tetrapolar set-up is that it requires a greater number of electrodes. However, the cost of using more electrodes diminishes rapidly with the number of body segments measured simultaneously, because the same couple of injecting electrodes (I1, I2) are employed for all the measured body segments.

**Figure 3:**
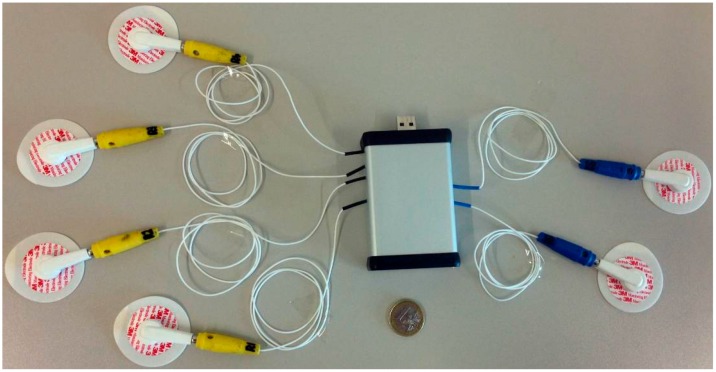
The realized prototype, with connectors for four sensing electrodes (yellow), two injecting electrodes (blue) and a USB port for data input/output with a personal computer; the tetrapolar electrode set-up allows using standard electrocardiographic disk electrodes.

The device determines Z*(f)* for each body segment at up to 10 frequencies *f*, roughly equispaced on a log-scale between 1 kHz and 796 kHz. The sampling rate of BIS measurements can reach up to 50 impedance spectra per second for each body segment. The system can operate uninterruptedly for several hours by recording the data locally on a Secure Digital card. The device can also be connected to a personal computer via a Universal Serial Bus (USB) 2.0 link (Figure 3) to monitor the data in real time. The USB connection also allows setting the acquisition parameters (number of frequencies, sampling rate). The device dimensions are as follows: depth = 8.5 cm, width = 5.5 cm, height = 2 cm. The weight is less than 100 g and the total power consumption is lower than 100 mW.

### 2.2. Experimental Application 

To illustrate the feasibility of our BIS system to track impedance changes associated with acute fluid shifts in three body segments, we investigated the effects of isolated dynamic lower limb exercise as well as the effects of sitting *versus* standing on Z*(f)*. The experimental protocol was designed to induce impedance variations by changing the volume of body fluids (that are conductive ionic solutions) in specific segments of the body. To this purpose, the protocol included a one-leg kicking exercise providing an increase in the local demand of blood flow in a single leg only. The increased blood flow would have accumulated a volume of fluids in the venous capacitance vessels of the exercising leg only, which would have been detected by our system. The protocol also included a change of posture from sit to stand, to induce a blood shift from both the legs.

The system was instrumented on a physically active male volunteer (age = 30 years; body height = 170 cm; body weight = 69 kg; fat mass percentage = 10.4%; maximum workload on cycle ergometer = 275 W; one-repetition maximum force, 1 RM = 75 kg).

**Figure 4:**
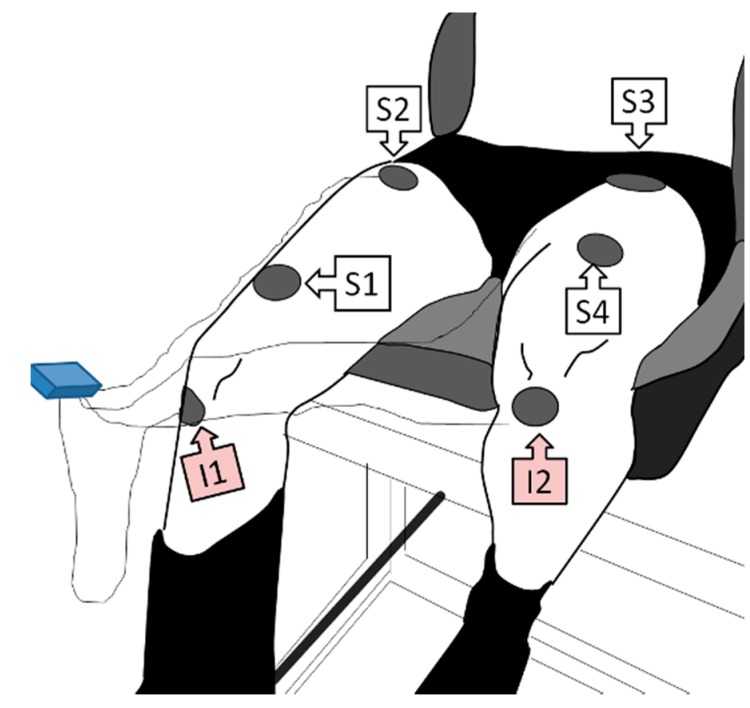
Example of subject instrumentation for BIS in the right thigh (S1,S2), in the pelvis (S2,S3) and in the left thigh (S3,S4).

The electrodes injecting the stimulus current were placed on the right (I1) and left (I2) knees, just below the tibiofemoral joint. The sensing electrodes were placed close to the distal (S1) and proximal (S2) endings of the rectus femoris muscle belly of the right thigh, and close to the proximal (S3) and distal (S4) endings of the contralateral muscle (Figure 4). Thus, the monitored body segments were the right thigh (S1,S2), the lower pelvis area (S2,S3) and the left thigh (S3,S4). 

For this application, the device was set to record impedances of the three body segments simultaneously every 6 s, at eight frequencies, *i.e.*, 4, 8, 17, 48, 128, 234, 488 and 796 kHz. These frequencies were selected to focus the impedance assessment mainly around the β-dispersion frequency band [18,19,20], as usually done in studies aimed at predicting extra- and intra-cellular water [21].

In the first part of the experiment, the volunteer sat on a one-legged, knee-extensor ergometer [22]. After a rest period of 7 min (“baseline” phase), the subject performed unilateral leg extensions with the dominant limb (right) at a rate of 60 extensions per minute. The leg-extension period consisted of an initial warm-up of a few minutes at 10 watts followed by 20 min of light exercise (“exercise” phase) at 25 watts (*i.e.*, less than 20% of the maximum workload). In the last two minutes of exercise the load increased to 50 watts. Exercise was followed by a recovery period of 20 min (“recovery” phase).

**Figure 5:**
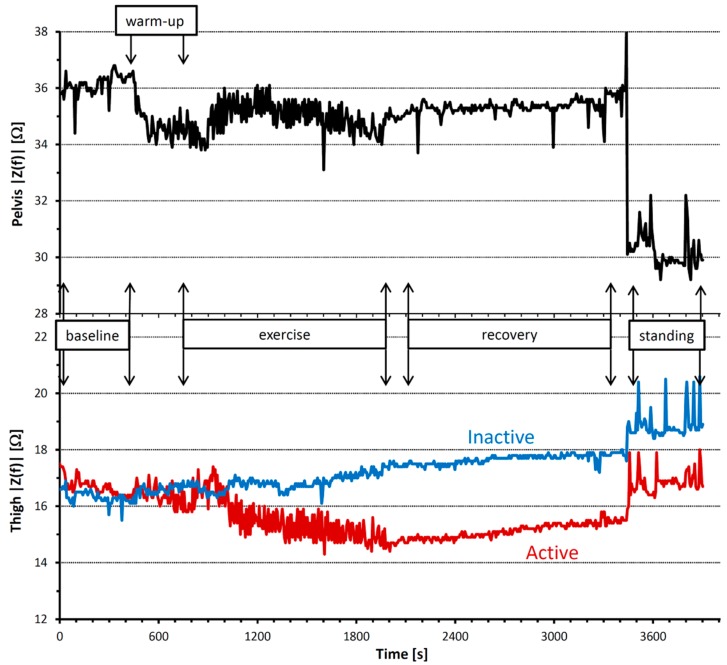
Profiles of |Z*(f)*| at *f* = 48 kHz in the pelvis area (**upper panel**) and in the active (red) and inactive (blue) thighs (**lower panel**) during the whole experimental session. Please note that the selected subperiods corresponding to “exercise”, “recovery” and “standing” conditions are spaced by a few minutes to exclude transitions phases from the analysis.

In the second part of the recording, the volunteer rested in standing position for 7 min (“standing” phase). In order to exclude transition phases from the analysis (Figure 5), the “recovery” phase was spaced by a few minutes from the “exercise” phase, and the “standing” phase was spaced by a few minutes from the instant in which the sit-to-stand change of the posture occurred.

During the whole experiment, skin temperatures of the right and left thighs were repeatedly measured with a non-contact infrared thermometer (826-T2, Testo spa, Settimo Milanese, Italy).

## 3. Results and Discussion

Figure 5 shows the time course of the impedance magnitude at 48 kHz in the three body segments. Impedance was similar in the two thighs at baseline, but markedly differed between the active and inactive leg during exercise. Its magnitude decreased in the active leg, with a fast variability component superimposed to the decreasing trend. By contrast, |*Z(f)*| increased in the inactive leg without showing the fast variability components. 

The decreasing impedance trend of the active thigh (red line) is likely due to the increased volume of blood accumulated in the active thigh, particularly in the capacitance vessels of the venous district, which follows the increased demand of blood flow in the upper leg. In contrast, an explanation for the fast variability component is that knee-extension muscles squeeze out blood from the thigh vessels during each contraction, thus decreasing the volume of fluids and conversely increasing the impedance. In addition, each knee extension may also have caused a synchronous change in impedance by modifying the cross-sectional areas of the contracting muscles [23].

In the pelvis area, impedance decreased at the start of the exercise warm-up. This behavior could be due to the fact that the pelvis is located just upstream of the active thigh and the increased blood flow demand for the exercising muscles may have also increased the blood volume in the neighboring area during warm-up. The increased impedance after warm-up in the pelvis and in the inactive thigh seems to reflect blood flow redistributions from these non-exercising segments to the active leg.

During exercise, pelvis impedance also demonstrated a fast variability component. It is assumed that part of the blood volume squeezed out by the contracting muscles of the active thigh was pushed into the pelvis, producing a fast periodic component similar to that observed in the active thigh. This fast variability disappeared when the muscle contractions stopped at the start of recovery, while the impedance difference between the inactive and active thighs persisted during the whole recovery period.

**Figure 6:**
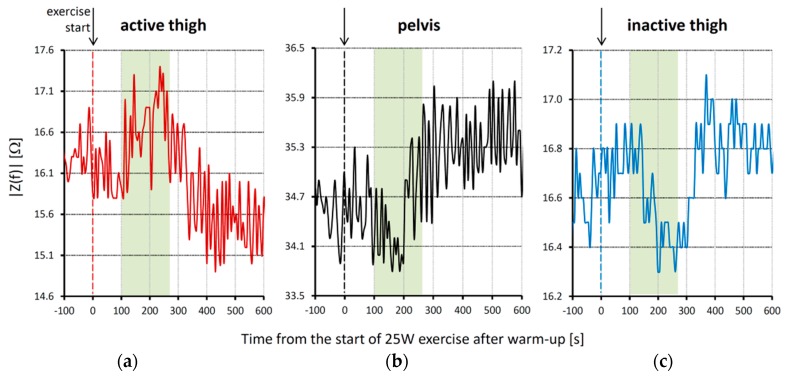
Changes of |Z*(f)*| at *f* = 48 kHz associated with the start of 25 W exercise after warm-up in three body districts (same experimental session of Figure 5): (**a**) active thigh; (**b**) pelvis area; (**c**) inactive thigh. The vertical lines at time zero indicate the start of 25 W exercise after warm-up. Please note the transient increase of impedance magnitude in the active thigh 100 s after the start of exercise, a simultaneous impedance decrease in the pelvis area, and a decrease of impedance in the inactive thigh that follows the impedance decrease in the pelvis. The green box highlights the time period corresponding to the transient impedance increase in the active thigh.

Our device appears to have the sufficient resolution and sensitivity needed for studying small acute circulatory responses during the first minutes following the start of exercise (note the enlargement of the transition from warm-up to exercise in Figure 6). These response are probably related to the mismatch between oxygen demand and oxygen supply by the blood flow in the working muscles. In fact, we can observe an increase of impedance magnitude in the active thigh that starts 100 s after the onset of exercise (Figure 6a). This increase is likely due to the working muscles’ contraction that occludes blood flow into the muscle and pushes out a substantial fraction of blood volume into the pelvis. In accordance with that, we also observe a temporary decrease of impedance in the pelvis area synchronous with the impedance increase in the active thigh (Figure 6b), which could correspond to the volume of blood shifted from the active thigh to the pelvis. It is known that an increased oxygen demand in a working muscle accumulates metabolites that stimulate chemically sensitive nerves in the muscular parenchyma and that, in turn, activates the muscle metaboreflex [24]. The efferent response of this reflex activation is an increase in the activity of the sympathetic nervous system that, on the one hand, induces an increase of cardiac output, aimed at increasing blood flow, and, on the other hand, constricts the vasculature in order to redirect the augmented flow of blood to the active muscles only. Vasoconstriction induced by the metaboreflex may have temporarily reduced the volume of blood in the pelvis, causing the rapid |Z*(f)*| increase 200 s after the onset of exercise (Figure 6b). Interestingly, the impedance of the active thigh starts to decrease about 50 s after the start of the impedance rise in the pelvis, suggesting that the decrease of impedance in the active thigh is, at least in part, a consequence of vascular vasoconstriction in the pelvis area. 

It is worth noting that a temporary decrease of impedance magnitude after the onset of exercise is detected not only in the pelvis, but also in the inactive thigh. Impedance of the inactive thigh starts decreasing about 50 s after the onset of the impedance rise observed in the active thigh. The lowest impedance values occur between 200 s and 300 s from the start of exercise, *i.e.*, after the |Z*(f)*| minimum in the pelvis. If the decrease of impedance magnitude in the inactive thigh derives from the volume of blood pushed from the active thigh, this would explain the delay from the decrease occurring in the pelvis area, which is more proximal to the active thigh. In any case, this finding is in line with the previously reported transient increase in femoral arterial blood flow of the non-exercising limb measured manually with a Doppler ultrasound instrument during one-legged exercise [25]. Therefore, the transient impedance changes we could observe continuously over time in these three body districts simultaneously after the start of exercise suggest that wearable BIS systems may support studies on the effects of activations of the metaboreflex, a physiological mechanism still difficult to investigate comprehensively in real field conditions.

**Figure 7:**
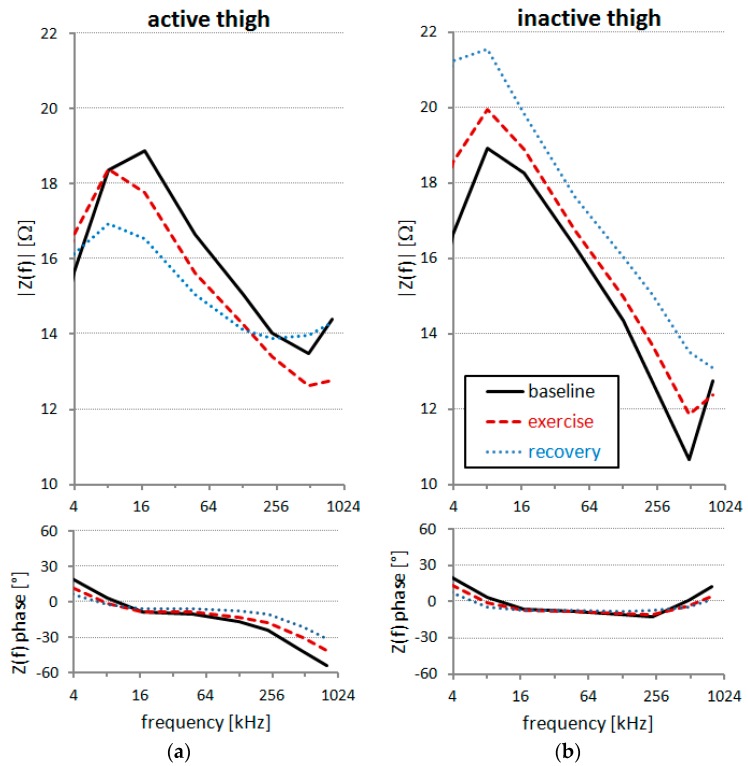
Z*(f)* during the leg-extension test in “baseline”, “exercise” and “recovery” periods in sitting position: (**a**) active thigh; (**b**) inactive thigh.

Impedance spectra of the two thighs, averaged in sitting position over “baseline”, “exercise” and “recovery” periods, showed opposite trends: |Z*(f)*| decreased from baseline to exercise and from exercise to recovery in the active thigh, while it increased from baseline to exercise and exercise to recovery in the inactive thigh (Figure 7). Interestingly, changes induced by exercise were more pronounced in the 16–64 kHz range for the active thigh, and in the 4–16 kHz range for the inactive thigh. At higher frequencies (≥488 kHz), small differences between the thighs are expected due to an inductive effect of stray capacitances affecting the more distal segment from the current injecting electrode (inactive thigh) [16]. 

**Figure 8:**
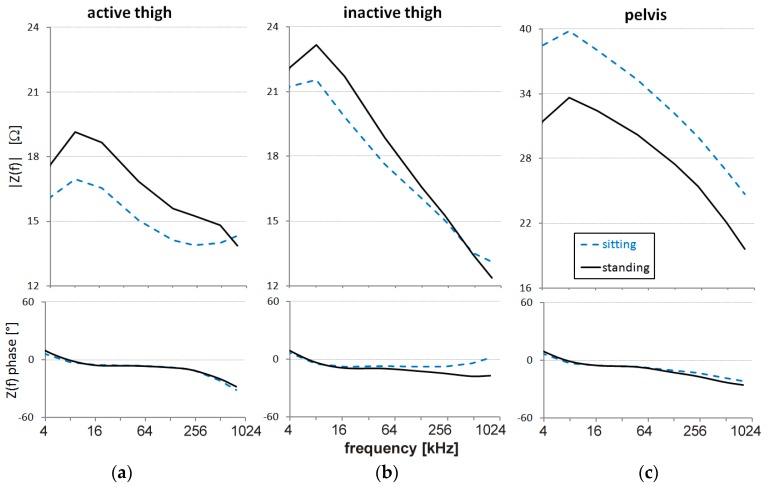
Modulus and phase of Z*(f)* in sitting and standing positions during recovery from exercise: (**a**) active thigh; (**b**) inactive thigh; (**c**) pelvis.

The effects of the sit-to-stand change of posture on impedance spectra are illustrated in Figure 8, comparing sitting during recovery with standing. The value of |*Z(f)*| increased from sitting to standing in both the thighs (Figure 8a,b), and the increase was more pronounced at the lower frequencies. By contrast, |*Z(f)*| of the pelvis area decreased markedly and uniformly over the whole frequency band from sitting to standing (Figure 8c).

The change of posture from sitting to standing suddenly increased |Z*(f)*| in both the thighs, likely because of the contraction of the anti-gravitational muscles pushing a fraction of blood volume out of the thighs and/or because of significant venous pooling in the distal part of the lower limbs. At the same time, the dramatic decrease of |Z*(f)*| in the pelvic region suggests that this body segment received a large volume of blood in the large splanchnic capacitance vessels.

Despite fluid shifts, changes in body temperature also should be considered among the possible physiological factors that may have affected impedance. Skin temperature increased slightly during the experimental protocol in the active thigh (+0.5 °C), after an initial drop at the onset of exercise, while it decreased in the inactive thigh (−2 °C), the difference between the thighs reaching 3 °C during exercise (see Table 1). Temperature may potentially affect BIS, and some authors reported decreased impedance when the skin temperature increased, hypothesizing the effects of temperature-induced changes in cutaneous blood flow (blood flow might not have directly influenced impedance, but vasodilation could have increased the vascular volume, reducing impedance) [26]. In addition, changes in skin temperature may also affect the skin-electrode interface [27]. However, other investigators concluded that the effects of skin temperature on impedance are insignificant [28] or minimal [29], or reported that temperature does not even affect the electrode-skin interface at all [30]. Interestingly, Cornish *et al.* (1998) [28] found that increased skin temperature, in fact, has an effect on impedance when a bipolar electrode arrangement is employed, but not when a tetrapolar electrode configuration is used: in this latter case, impedance changed by less than 2% when skin temperature changed by 15 °C. We may therefore assume that effects of body temperature on impedance are only marginal with the tetrapolar electrode set-up that was used with our device. In particular, given the small differences in skin temperature between the thighs and between the start and end of exercise (see Table 1), we may exclude substantial effects of body temperature on our present findings. Finally, sweating might be another factor influencing the BIS, but because of the relatively light exercise load, and the small increase of temperature, sweating did not occur during exercise. In any case, the tetrapolar electrodes arrangement has also been shown to be largely insensitive to the effects of sweating [28].

## 4. Conclusions

The prototype we presented has the lightness, wearability and unobtrusiveness that “real field” studies of sports, aerospace and rehabilitation medicine require. The use of a DSP and of proper stimulation waveforms allows monitoring more segments simultaneously and continuously for extended periods. This makes it possible to quantify detailed alterations in the composition of specific body segments in physiological and clinical settings, where the use of traditional BIS systems is presently limited.

The exercise test clearly illustrated the unique information provided by such types of wearable devices. We showed that *Z(f)* can be quantified over different time scales, from the fast dynamics of muscle contraction or sit-to-stand postural shift, to the long-term dynamics of exercise recovery. Monitoring different body segments simultaneously allowed detecting shifts of blood volumes among contiguous segments (as between active and inactive muscles). This would not be possible with traditional BIS devices, designed to monitor one body segment at a time. Moreover, we reported that the one-leg extension exercise affected components at different frequencies on the active and inactive thighs, suggesting that the blood shift between legs is associated with different ratios of intra-cellular and extra-cellular fluid compartments. This finding would not be observed with traditional mono-frequency instruments. 

Some intrinsic limitations of BIS methodology also have to be considered when using our system. First, the interpretation of *Z(f)* variations in terms of changes in tissue composition of a given body segment is based on statistical population models, which might be inaccurate in specific cases (see Appendix A). Second, these models assume that body segments are composed by parallel cylinders of homogeneous conductors, oversimplifying the real tissue structure of large body segments. However, this limitation is mitigated by splitting individual body districts in a series of smaller segments, an approach that multi-segmental devices such as our prototype can easily realize, revealing the true potential of BIS. Finally, we would like to acknowledge a general limitation of our study. The analysis was conducted as a pilot study on a single healthy, trained and lean male volunteer. We could easily track impedance changes during the whole protocol in this volunteer. However, the detectability of BIS changes may depend on physical characteristics (such as regional adiposity) that may be different in specific groups of patients. While we demonstrated the potential feasibility of this approach, further studies on larger populations are therefore required to confirm its validity in various populations and settings. 

## Figures and Tables

**Figure A1:**
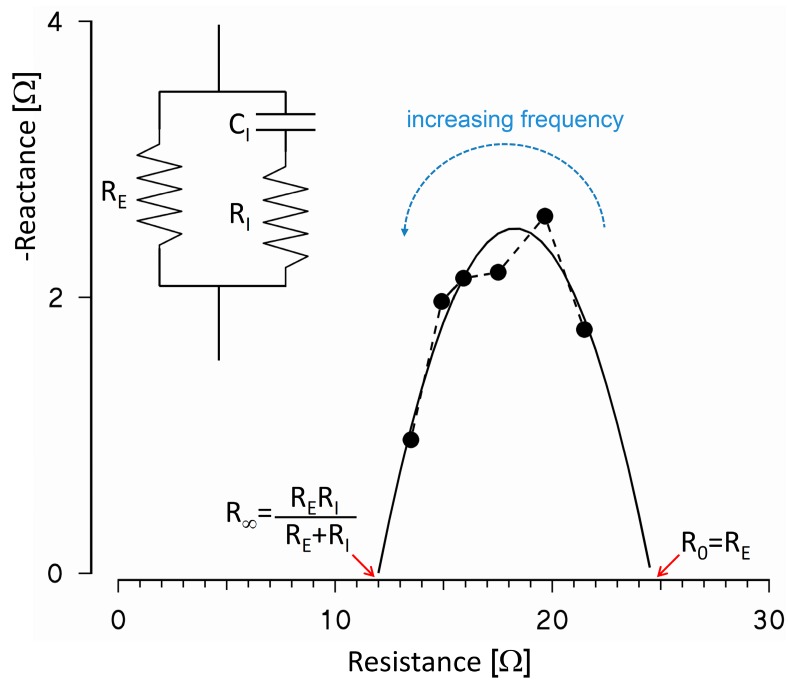
Resistance and reactance of *Z(f)* values in the β-dispersion range (solid circles) fitting the simplified equivalent electric circuit represented by the resistance R_E_ of extra-cellular fluids in parallel with the membrane capacitance C_I_ and the resistance R_I_ of intra-cellular fluids (see inset); the mathematical curve that interpolates the measured values crosses the resistance axis at R_0_ (corresponding to the model resistance when a DC current is injected) and at R_∞_ (corresponding to the equivalent resistance of the model at infinite frequency). The example shows *Z(f)* data measured in the inactive thigh during recovery in sitting position.

**Table 1:** Skin temperature in active and inactive thighs during the whole experimental protocol.

Time	Condition	Thigh Temperature (°C)	
		Active	Inactive
400 s	Baseline	32.5	32.5
750 s	warm-up (10W)	32.5	31.5
1250 s	exercise (25W)	31.0	29.0
1500 s	exercise (25W)	32.0	29.0
1800 s	exercise (25W)	32.5	29.5
1950 s	exercise (50W)	33.0	30.5
2550 s	Recovery	33.0	31.0
3900 s	Standing	33.0	30.5

## Supplementary Files

Supplementary File 1

## Appendix A: From BIS Measures to the Determination of Body Fluids Volumes

In the 1980s, biophysical models of living tissues suggested the possibility to quantify distributions of intra-cellular and extra-cellular fluids in individual limbs [31], and since the 1990s, BIS has been proposed as a noninvasive alternative to radio-isotopic tracer dilution techniques for assessing fluid volumes of the whole body [3]. BIS methods fit *Z(f)* values measured at different frequencies *f* to a biophysical model of the whole body, or of one of its segments. Proposed methods differ in terms of the adopted biophysical model and the procedures for data fitting. For a review on these models and for an evaluation of their agreement in estimating extra-cellular, intra-cellular and total body water, readers are referred to [3].

Briefly, BIS methods aimed at determining body fluid volumes focus on the frequency band defined as β-dispersion, which depends on the type of tissues [18] and may range between 1 kHz and 1 MHz. The β-dispersion reflects the dielectric properties of extra- and intra-cellular electrolytes as well as of cell membranes. An electric current injected in body tissues can flow around the cells through the extra-cellular fluids (an ion solution with known resistivity, ρ_E_), or can cross the cell membrane, flowing through the intra-cellular medium (whose resistivity, ρ_I_, depends upon the type of cell). The equivalent electric circuit of the extra-cellular fluids is a resistance, R_E_; the equivalent circuit of a cell is the series of the electric components describing the membrane and the intra-cellular medium. The membrane can be represented as the capacitance, C_I_, in parallel with a high resistance, and the intra-cellular medium as a resistance, R_I_. The electric model of the whole tissue can be highly simplified by ignoring the high resistance of cell membranes, and by modeling a tissue composed by several cells as the equivalent electric circuit of a single cell in parallel with the extra-cellular fluids, as depicted in Figure A1 (inset). At the lower frequencies, the electric conductivity depends mainly on R_E_ because of the cell membrane capacitance (in particular, a direct current cannot flow through the capacitor C_I_). By contrast, at higher frequencies, the injected current can cross the cell membranes and, when the frequency approaches infinity, the capacitor does not hinder the current, which depends only on R_E_ and R_I_ connected in parallel.

However, this simplified model cannot be assumed to be valid for frequencies falling outside the range of β-dispersion, where other electric phenomena (such as ion diffusion or dipolar moments) may play a role. This means that R_E_ cannot be measured by injecting a DC current, or that the parallel between R_E_ and R_I_ cannot be measured by injecting currents at extremely high frequencies. By contrast, these model parameters should be derived by mathematically interpolating resistances (*i.e.*, the real part of *Z(f)*) and reactances (*i.e.*, the imaginary part of *Z(f)*) measured by BIS, as in the example of Figure A1.

When R_E_ is estimated as R_0_ after *Z(f)* data fitting, the volume of extra-cellular water, *V_E_*, in a given body segment can be assessed easily by assuming that the body segment is a cylinder of length *l* and volume *V.* In this case, the electric resistance due to the extra-cellular fluids is:

(A1) $$R_{E}=\rho \frac{l^{2}}{V}$$

where the resistivity *ρ* is that of non-conducting tissues imbedded in the extra-cellular ionic solution of known resistivity *ρ*_E_. Hanai’s mixture conductivity theory allows obtaining *ρ* from *ρ*_E_ as:

(A2) $$\rho =\frac{\rho_{E}}{d^{3/2}}$$

where *d* is the volume fraction of the conducting tissues. At low frequencies, only the extra-cellular fluids may conduct the injected current and:

(A3) $$d=\frac{V_{E}}{V}$$

Therefore, combining Equations (A1)–(A3), one may derive the following expression:

(A4) $$V_{E}={\left(\rho_{E}\frac{V^{1/2}l^{2}}{R_{E}}\right)}^{2/3}$$

The derivation of intra-cellular water volume, *V_I_*, or of total water volume, *V_E_* *+ V_I_*, is more critical, and different approaches were proposed in the literature. Following [32], the first step is to derive *R_I_* from *R_E_* and *R_∞_* as:

(A5) $$R_{I}={\left(\frac{1}{R_{\infty }}-\frac{1}{R_{E}}\right)}^{-1}$$

and *V_I_* by solving:

(A6) $${\left(1+\frac{V_{I}}{V_{E}}\right)}^{5/2}=\frac{R_{E}+R_{I}}{R_{I}}\left(1+\frac{\rho_{I}}{\rho_{E}}\frac{V_{I}}{V_{E}}\right)$$

However, Equation (A6) requires the knowledge of *ρ_I_* or of the resistivity ratio rr = *ρ_I_*/*ρ_E_*. These values may depend on the specific body segment. Moreover, discrepant results are reported in the literature. For the whole body, Van Loan *et al.* proposed rr = 3.82 for men, rr = 3.40 for women [32]. These values were considered to be too low by De Lorenzo *et al.* [21], who proposed rr = 6.76 for men, rr = 6.79 for women, and too high by Ellis and Wong [33], who proposed rr = 3.03 for men, rr = 2.69 for women.

A final consideration regards the appropriateness of modeling individual body segments, such as a limb, as cylindrical volumes of homogeneous composition. A more sophisticated model, which takes into account the non-homogeneous nature of body segments, represents each segment as the equivalent circuits, in parallel, of four concentric cylinders of skin, fat, intra- and extra-cellular fluids, and bones, each cylinder being characterized by its own resistance and capacitance [34]. Alternatively to the cylindrical shape, it has been also proposed to represent limb geometry more realistically as truncated-cone elements [35].
