# Supplementary material for: Wearable Multi-Frequency and Multi-Segment Bioelectrical Impedance Spectroscopy for Unobtrusively Tracking Body Fluid Shifts during Physical Activity in Real-Field Applications: A Preliminary Study
Source: Sensors (Basel). 2016 May 11;16(5):673. doi: 10.3390/s16050673 (PMC4883364; doi:10.3390/s16050673)
Supplement: Supplementary File 1 [file sensors-16-00673-s001.pdf]

Supplementary Materials: Wearable Multi-Frequency and Multi-Segment Bioelectrical Impedance Spectroscopy for Unobtrusively Tracking Body Fluid Shifts during Physical Activity in Real-Field Applications: A Preliminary Study

Federica Villa, Alessandro Magnani, Martina A. Maggioni, Alexander Stahn, Susanna Rampichini, Giampiero Merati and Paolo Castiglioni

The following table lists the more popular devices on the market for assessing Bioelectrical Impedance Spectroscopy, BIS (*i.e.*, body impedance analysis performed by considering more than one frequency). Few devices only provide measures at different segments simultaneously, and in this case such devices consider only the limbs and the trunk as measured segments. None of the listed systems is wearable. When the same manufacturer offers different products, only the more complete model is listed.

Table S1. Characteristics of the main BIS devices currently found in the market.

Model	Number of Frequencies	Range	Multi Segment	Wearable
Impedimed SFB7 [1]	256	4–1000 kHz	No	No
XiTRON Hydra 4200 [2]	50	5–1000 kHz	No	No
Bodystat multiscan 5000 [3]	50	5–1000 kHz	No	No
Bodycom MF HEXA [4]	6	5–250 kHz	No	No
Fresenius Medical Care BCM [5]	50	5–1000 kHz	No	No
GE Healthcare Inbody 720 [6]	6	1–1000 kHz	Yes	No
Tanita MC-980UPlus [7]	3	5–250 kHz	Yes	No
Rice Lake D1000-3 [8]	3	5–250 kHz	Yes	No

References

1. Imp™ SFB7. Available online: https://www.impedimed.com/wp-content/products/SFB7/SFB7_CA_Brochure.pdf (accessed on 5 May 2016).
2. XiTRON Hydra 4200 Specifications. Available online: http://www.xitrontech.com/assets/002/5853.pdf (accessed on 5 May 2016).
3. Clinical Practice: Multiscan 5000. Available online: http://www.bodystat.com/pdf/spreads/multiscan.pdf (accessed on 5 May 2016).
4. Bodycomp MF HEXA. Available online: http://www.akern.com/images/DP_BC_MF_HEXA_ITA_Rev0.pdf (accessed on 5 May 2016).
5. BCM—Body Composition Monitor. Available online: http://www.bcm-fresenius.com/16.htm (accessed on 5 May 2016).
6. Lunar InBody720 Spec Sheet. Available online: http://www3.gehealthcare.com.sg/~/media/documents/us-global/products/metabolic-health/product%20spec%20sheets/inbody/gehealthcare-inbody-720-productspec.pdf (accessed on 5 May 2016).
7. MC-980Uplus Multi Frequency Segmental Body Composition Analyzer. Available online: http://www.tanita.com/en/mc-980uplus (accessed on 5 May 2016).
8. Rice Lake D1000-3. Available online: https://www.ricelake.com/docs/prodinfo/rlws_catalog/Individual/medical/MS_Body_Comp_Full_Consult.pdf (accessed on 5 May 2016).
